# Clinical implementation of a neonatal seizure detection algorithm

**DOI:** 10.1016/j.dss.2014.12.006

**Published:** 2015-02

**Authors:** Andriy Temko, William Marnane, Geraldine Boylan, Gordon Lightbody

**Affiliations:** a Neonatal Brain Research Group, INFANT Research Centre, Dept. Electrical and Electronic Engineering, University College Cork, Cork, Ireland; b Neonatal Brain Research Group, INFANT Research Centre, Dept. Pediatrics and Child Health, University College Cork, Cork, Ireland

**Keywords:** Neonatal seizure detection, EEG, Visualization, Audification, Clinical interface, Decision making

## Abstract

Technologies for automated detection of neonatal seizures are gradually moving towards cot-side implementation. The aim of this paper is to present different ways to visualize the output of a neonatal seizure detection system and analyse their influence on performance in a clinical environment. Three different ways to visualize the detector output are considered: a binary output, a probabilistic trace, and a spatio-temporal colormap of seizure observability. As an alternative to visual aids, audified neonatal EEG is also considered. Additionally, a survey on the usefulness and accuracy of the presented methods has been performed among clinical personnel. The main advantages and disadvantages of the presented methods are discussed. The connection between information visualization and different methods to compute conventional metrics is established. The results of the visualization methods along with the system validation results indicate that the developed neonatal seizure detector with its current level of performance would unambiguously be of benefit to clinicians as a decision support system. The results of the survey suggest that a suitable way to visualize the output of neonatal seizure detection systems in a clinical environment is a combination of a binary output and a probabilistic trace. The main healthcare benefits of the tool are outlined. The decision support system with the chosen visualization interface is currently undergoing pre-market European multi-centre clinical investigation to support its regulatory approval and clinical adoption.

## Highlights

• We present different ways to visualize the output of a neonatal seizure detector.
• We establish the connection between information visualization and task metrics.
• We report the results of the survey of clinicians on a suitable visualization method.
• The developed DSS is currently undergoing multi-centre clinical trial.

## 1 Introduction

Neonatal seizures are the most common neurological emergency in the neonate and are a serious concern for clinicians and parents worldwide [1]. Only about one third of all neonatal seizures are clinically visible [2] and many remain undetected in the busy Neonatal Intensive Care Unit (NICU) environment. The only method available to detect all neonatal seizures accurately is continuous multi-channel EEG monitoring. Interpretation of neonatal EEG requires a neurophysiologist or paediatric neurologist with specific expertise in neonatal EEG. This expertise is not available on a 24 h basis, 7 days a week [3]. To fill the gap in the lack of availability of experts, clinical staff in the NICU are using a simpler form of EEG monitoring, called amplitude integrated EEG or aEEG [4]. Amplitude integrated EEG is a logarithmically-scaled, temporally-smoothed and compressed display of EEG which is usually computed from two EEG channels, one from each hemisphere. Despite the fact that many short and focal neonatal seizures are undetectable with aEEG and interobserver agreement is poor [5], aEEG currently serves as a trade-off between very inaccurate clinical detection of seizures and very accurate but scarcely available neurophysiologic expertise, and thus is widely adopted worldwide in the NICU [3].

As an alternative to aEEG usage, many groups in the world are working to develop algorithms for automated detection of neonatal seizures on continuous multi-channel EEG. An automated decision support system (DSS) that could detect and annotate seizures on the neonatal EEG would be extremely useful for clinicians in the NICU [44]. A number of methods have been previously proposed but to date their transition to clinical use has been limited due to: (i) the proof of concept nature of the work performed, which involved carefully selected short-duration EEG segments [6–9]; (ii) an unrealistic validation regime such as testing on training data or excluding the worst performing records [10–12]; and (iii) the provision of algorithm performance which is currently unacceptable in a clinical setting [13–17].

There are two key directions in automated neonatal seizure detection. The first follows analytical learning principles [18] and focuses on the creation of a set of heuristic rules and thresholds from clinical prior knowledge [6–8,10,12–15]. The resultant detectors analyse EEG using a small number of the descriptors from which a decision is made using empirically derived thresholds. Binary decisions are obtained with this approach. The second approach relies on inductive learning [18] and utilizes model-based parameterization [9,16] or statistical classifier based methods [11,19,20], which employ elements of machine learning to classify a set of features using a data-driven decision rule. This approach is capable of outputting continuous confidence of decisions such as probability of seizure.

**Fig. 1:**
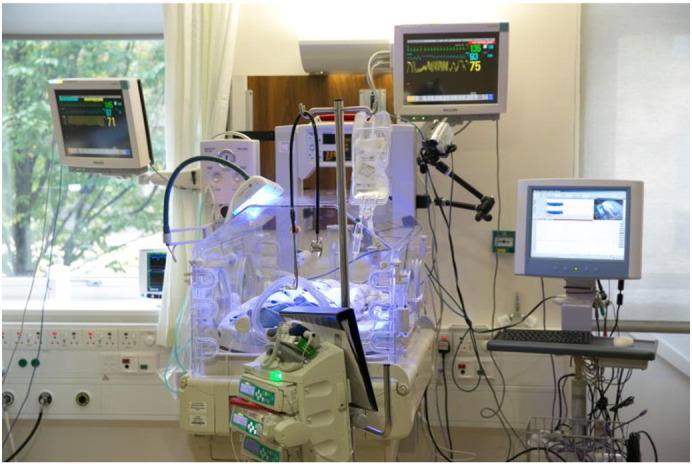
Clinical environment in NICU with EEG monitoring system on the right.

Our group has recently developed [19,21], validated [20] and patented [22] an accurate and robust real-time neonatal seizure detection system combining both of these approaches. In order to have the system used at the cot-side, as well as to help achieve regulatory approval, we need to identify the most intuitive and synergetic way to convey the system output information to neonatal caregivers. In this context, when the developed technology approaches cot-side implementation, it becomes important to build a viable interface between the new engineering component and established medical environments [23–25]. The NICU environment (Fig. 1) already has plenty of technologies, including a number of physiological monitors; adding yet another ‘technology’ becomes a challenging task.

In this study, we propose and examine 3 different ways to visualize the output of an automated neonatal seizure detector: a binary output, a probabilistic output and a spatio-temporal colormap output. Additionally, the algorithm-driven audification of neonatal EEG is also explored as an alternative to a visual output. Five neonatologists with experience in interpreting the cotside EEG from the second largest maternity hospital in Europe (Cork University Maternity Hospital) were surveyed over approximately 1 h, answering over 100 questions, and the survey results are also reported in this work.

The paper is organized as follows: Section 2 briefly describes the neonatal seizure detection system developed by the group. Section 3 describes 3 different ways to visualize the system output information along with audification of neonatal EEG. A link between the ways that the metrics are computed and the system output is visualized are established in Section 4. Section 5 presents and discusses the survey results. Section 6 introduces the chosen interface for the developed DSS which is currently undergoing pre-market European multi-centre clinical investigation to support its regulatory approval and clinical adoption. Section 7 links the study to the theory of DSS. Economic benefits of the developed technology are outlined in Section 8. Our expectations from the results of the clinical trial are given in Section 9. Conclusions are drawn in Section 10.

## 2 Neonatal seizure detector

**Fig. 2:**
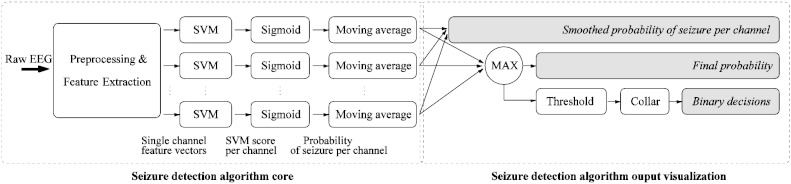
Neonatal seizure detection system diagram.

The developed automated neonatal seizure detection system is shown in Fig. 2. A video EEG machine was used to record multi-channel EEG using the 10–20 system of electrode placement modified for neonates. The following 8 EEG channels in bipolar pairs are used to feed the EEG data into the system: F4–C4, C4–O2, F3–C3, C3–O1, T4–C4, C4–Cz, Cz–C3 and C3–T3. It has been shown that frequencies of neonatal EEG seizures range between 0.5 and 13 Hz and the dominant frequencies of seizures vary between 0.5 and 6 Hz [26]. The EEG from the 8 channels is downsampled to 32 Hz with an anti-aliasing filter set at 12.8 Hz. The EEG is then split into 8 s epochs with 50% overlap between epochs. The most recent recommendations by the International Federation of Clinical Neurophysiology [27] suggest that 5 s is the minimum seizure duration if the background EEG is normal and 10 s if the background EEG is abnormal. A window length of 8 s was chosen given that hypoxic ischemic encephalopathy (HIE) is the commonest cause of seizure in the full term neonate and the background EEG is always abnormal in those with seizures. This window length would also prevent short duration seizure-like events (e.g. brief intermittent rhythmic discharges) being incorrectly detected as seizure events. A long feature vector which consists of fifty-five features is extracted from each epoch. The features are designed to convey both time and frequency domain characteristics as well as information theory based parameters.

A Support Vector Machine (SVM) classifier is trained on data which are normalized anisotropically by subtracting the mean and dividing by the standard deviation to assure commensurability of the various features. This normalizing template is then applied to the testing data. The obtained classifier is applied separately to each channel of the testing data as neonatal seizures can be localized to a single EEG channel. The output of the SVM is converted to probability-like values with a sigmoid function [28]. The probabilistic output is then time-wise smoothed with a moving average filter. Detailed information on the system can be found in [19].

Several important enhancements of the developed system have recently been investigated. A wider feature set which included spectral slope features from speech recognition has been examined in [29]. A Gaussian mixture model classifier has been developed in [30] and contrasted to SVM with the classifier combination performed in [31]. Adaptive spatial weighting of EEG channels based on the statistics of spatial neonatal seizure distributions has been introduced in [32]. Similarly, temporal weighting of the probabilistic output of the classifier based on the statistically most likely locations of neonatal seizures since the time of birth has been introduced in [21]. The short term seizure event context has been shown to increase the robustness of the detector to the seizure-like artefacts, in particular the respiration artefact [33].

The developed system has been validated in [20,21,33] using leave-one-patient-out (LOO) cross validation which is known to provide the least biased assessment of performance. This was achieved using a large clinical dataset, comprising long unedited multi-channel EEG recordings from 18 neonates with seizures and 20 neonates without seizures, totalling 1479 h of multi-channel EEG in duration and with 1389 seizures. Subsequently, the system was independently validated in [34] on a separate dataset of 41 neonates (full-term HIE, 7 with seizures, 377 seizures) and, more recently in [33], on a larger randomised dataset comprising 51 full-term neonates with HIE (24 with seizures, 1142 seizures, totalling 2540 h of multi-channel in duration). In both cases, retrospectively with LOO cross validation and using prospective datasets, similar levels of performance were achieved as measured by the mean area under the receiver operating characteristics curve (AUC) with 95.4% in [34], 96.1% in [33] and 96.7% in [21].

The system is currently undergoing a pre-market European multi-centre clinical investigation. The chosen way to visualize the system output in a clinical environment should maximise the synergy between the existing clinical practice and the support provided by the developed tool.

It is possible to see from Fig. 2 that the system can output multiple probabilistic traces, one per each channel. The maximum of the averaged probabilities across all channels can be computed to represent the final support of a seizure resulting in a single overall probabilistic trace. This probabilistic trace can be compared with a threshold to produce a trace of binary decisions: 1 for seizure and 0 for non-seizure. The ‘collar’ technique is applied last — every seizure decision is extended from either side to account for the delay introduced by the moving average smoothing and to compensate for possible difficulties in detecting pre-seizure and post-seizure parts. This will result in the binary decision output. In the next section, the main advantages and disadvantages of these representations will be discussed.

## 3 Visualization methods

### 3.1 Amplitude-integrated EEG

**Fig. 3:**
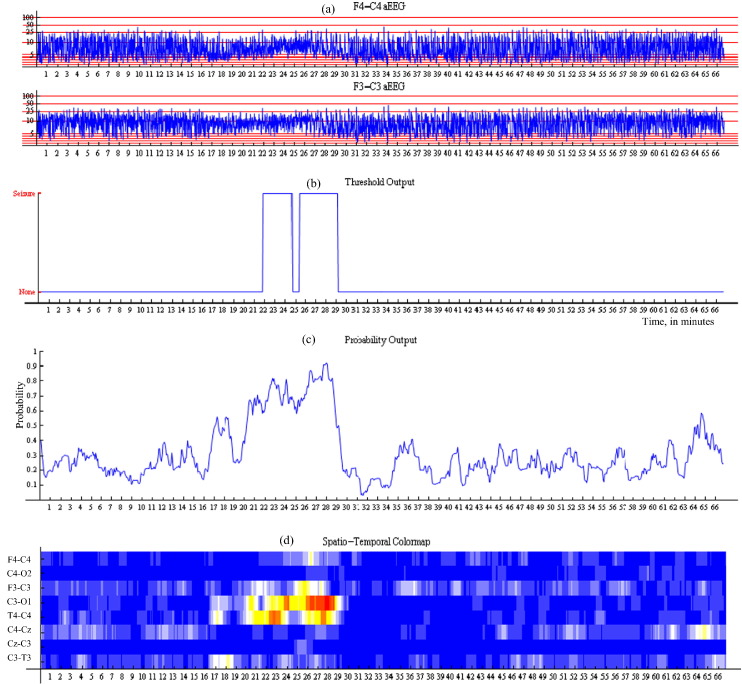
Example of visualization of the output of the neonatal seizure detection system for 66 m of EEG. Plot (a) indicates aEEG channels, plot (b) — binary output at a threshold of 0.7, plot (c) — probabilistic output, and plot (d) — spatio-temporal map. Seizure onset and offset are annotated as 16 min 37 s–30 min 05 s. Best viewed in colour.

The amplitude-integrated EEG is widely used in NICUs. There have been numerous studies that report low sensitivity of this tool for neonatal seizure detection and its inappropriateness for use in the neonatal population in general [5,42]. Technically, the filtered EEG signal is first rectified i.e. negative voltages are converted to positive values. The amplitudes are then smoothed using a moving average filter and the final result is plotted on a semi-logarithmic scale which is linear from 0 to 10 μV, and logarithmic from 10 to 100 μV. The aEEG emphasises the amplitude of the EEG signal [35]. Interpretation of aEEG is primarily based on pattern recognition and experience of the user is important. The maximum and minimum peak-to-peak amplitudes of the EEG signal are displayed to indicate the variance in aEEG amplitudes. Typically, an increase in the lower border of the aEEG trace is representative of seizures as shown in Fig. 3(a).

### 3.2 Binary output

The binary output as shown Fig. 3(b) is the simplest way to convey system output information to neonatal healthcare professionals. It displays 1 when there is a seizure according to the automated system and 0 when there is none and corresponds to the ‘binary decision’ in Fig. 2. As mentioned in the introduction, most reported systems that are based on a set of rules and thresholds are explicitly designed to provide the binary output. At the same time, the binary output can also be obtained from the systems that provide the continuous probabilistic output. The binary output is intuitive and there is no need in training for interpretation.

The need for a chosen threshold is a clear disadvantage. The threshold on its own does not result in bad or good performance; it is a point on the performance curve which merely defines a trade-off between the two competing metrics — the rate of correctly detected seizures and the rate of false alarms. For the task of neonatal seizure detection, many variables such as the medical/economic/social costs/risks of being falsely treated as a seizure patient or being falsely considered as a non-seizure patient must be incorporated to find an optimal threshold. The optimal threshold is a slope on the operating curve and finding it is a difficult task in its own right [36]. Similarly, the binary form does not contribute any confidence to the decision making process. It is thus not possible to derive how much in excess of the chosen threshold the system output was.

### 3.3 Probabilistic output

The problem of decision confidence can be addressed by visualizing the probability trace instead of the binary output, as seen in Fig. 3(c). The probability rises when seizure activity is suspected in the EEG. Thus, the probabilistic trace provides a measure of the confidence of the decision. With the probabilistic output, an additional temporal context dimension is introduced into the decision making — it is possible to perceive the level of increase of the current probabilistic activity over the past probabilistic activity. The probabilistic system output corresponds to ‘Final probability’ in Fig. 2.

Although it seems to make sense at first to provide this information to a neonatal health care professional, one might argue that the disadvantage of the probabilistic method is that the healthcare professional will have to look at all of the suggested possible seizures, even those with low confidence. Thus, the system can only support a decision that is already made. In contrast, if the physician decides not to look at seizures with lower than, say, 70% confidence rating, then a threshold has just been chosen similarly to the binary output case.

### 3.4 Spatio-temporal colormap

It is well known that seizures evolve both temporally and spatially. The spatial component is not included with the previous two methods. Although it is possible to generate 8 probabilistic traces from ‘Smoothed probability of seizure per channel’ in Fig. 2, it would be difficult to display them compactly and intuitively for healthcare professionals. A colormap to convey this information is proposed as seen in Fig. 3(d). The colormap is designed to range from cold blue (probability 0) to warm red (probability 1) though neutral white (probability 0.5). In total, 10 different colours are used to simplify the interpretation.

The colormap allows the observation of both temporal and spatial contexts of the confidence output of the system. Thus, the information content is the highest among the discussed methods. The interpretation becomes difficult and the clinical personnel will need to be pre-trained to be able to correctly interpret the colormap.

### 3.5 Audified neonatal EEG

It is believed that human hearing input is better than the visual input when it comes to assessing both the spatial and temporal evolution of the frequency characteristics of a signal. Hearing is flexible, low-cost and fast and there are a range of available algorithms that can synthesize the sound from data in order to make specific features within the data perceptible. Sonification or auditory display of signals naturally extends visualization.

**Fig. 4:**
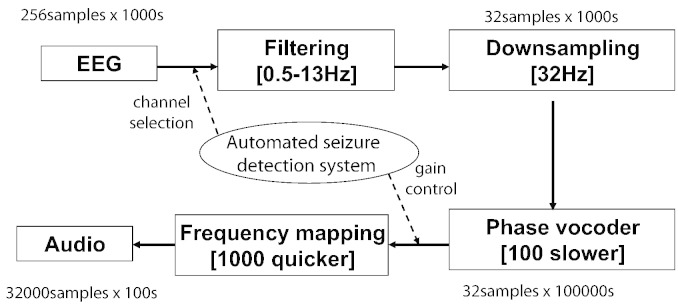
A flowchart for audification of 1000 s of neonatal EEG.

Human EEG has previously been audified for the purpose of the detection of epilepsy in adults in [37,38]. In this work, neonatal EEG is audified to assess its usefulness for the detection of neonatal seizures. The process is outlined in Fig. 4 with an example of 1000 s of EEG as an input. First, the same pre-processing steps are applied to EEG as in the seizure detector. Then, the EEG is passed through the phase vocoder [39,40] to change the temporal characteristics of the signal while retaining its short-time spectral characteristics. This process intuitively corresponds to stretching the time-base of a signal spectrogram. The signal sampled with 32 Hz is thus slowed down by a factor of 100 by the phase vocoder. It is then saved with 32 kHz sampling frequency. This corresponds to the frequency mapping of the original range of 0.5–13 Hz to the new range of 0.5–13 kHz so that the most dominant frequencies of seizure 0.5–6 Hz are mapped to the most sensible audible range, in particular to the range of human scream 3–4 kHz. The EEG audification technique allows for speeding up the EEG real-time play-back, in our case by a factor of 10 allowing 1 h of EEG to be played in roughly 6 min.

The resultant audio signal is made stereo, with left/right channels corresponding to left/right brain hemispheres. In contrast to EEG audification in [37,38], the automated seizure detection algorithm is used here to select a channel from each hemisphere with the highest cumulative seizure probability. Moreover, the signal gain is controlled by the probabilistic output of the system, thus accentuating suspicious EEG segments.

## 4 Visualization and metrics

A few metrics are commonly used to quantify the performance of the neonatal seizure detector. The metrics can be based on patients (whether a patient had at least one seizure or none), events (seizure events or false detections) and epochs (seizure burden). For instance, sensitivity can refer to the accuracy of detecting seizure patients, accuracy of detecting seizure events or temporal precision of detected seizure onsets and offsets. The competing metric is specificity or 1—specificity which measures the rate of false detections such as falsely detected seizure patients, the number of false seizure detections per hour or the amount of falsely detected seizure activity in time. Several differences in how these metrics should be computed for online, in contrast to offline systems, for seizure detection have been addressed in [41]. In this section, the connection between the computed metrics and the system output visualization is established.

Consider a choice of *N* threshold values such that *θ*_*i*_ ∈ {*θ*_1_, …, *θ*_*N*_}. If there are *M* testing patients in the dataset, then a specificity matrix *SP* = (*SP*_*ij*_) ∈ *R*^*N* × *M*^ and sensitivity matrix *SE* = (*SE*_*ij*_) ∈ *R*^*N* × *M*^ can be produced where *SP_ij_* (*SE_ij_*) is the specificity (sensitivity) results for the *j*th patient with the threshold choice *θ_i_*. The final metric (AUC) can be computed in two different ways. First, the AUC can be computed for each patient independently (for instance, using an average of a number of trapezoidal approximations) and then averaged across patients:

(1) $$AUC=\frac{1}{M}\sum_{j=1}^{M}\sum_{i=2}^{N}\left(SP_{i,j}-SP_{i-1,j}\right)\frac{\left(SE_{i,j}+SE_{i-1,j}\right)}{2}\text{.}$$

Alternatively, specificity and sensitivity values can first be averaged across patients and then the final AUC is computed as:

(2) $$AUC=\sum_{i=2}^{N}\left({\bar{SP}}_{i}-{\bar{SP}}_{i-1}\right)\frac{\left({\bar{SE}}_{i}+{\bar{SE}}_{i-1}\right)}{2}$$

where

(3) $$\begin{array}{ll} {\bar{SE}}_{i}=\frac{1}{M}\sum_{j=1}^{M}SE_{i,j}, & {\bar{SP}}_{i}=\frac{1}{M}\sum_{j=1}^{M}SP_{i,j} \end{array}\text{.}$$

With the latter method, the reported performance is meaningful only if the system output is visualized in the binary form. This happens because the sensitivity and specificity values are threshold-wise averaged across patients (Eq. (3)). Thus, the final AUC summarises the performance that the system will achieve with a particular threshold for all patients. Therefore, if the system is designed to output binary values, the correct way to report the performance of such a system is by averaging its sensitivity and specificity across patients before computing the AUC.

In contrast the calculation of the AUC for each patient separately and averaging them, summarises not the performance of the system for a particular threshold for all patients but rather a discriminability of the probabilistic output of the seizure detection system for each patient which is averaged across all patients.

**Fig. 5:**
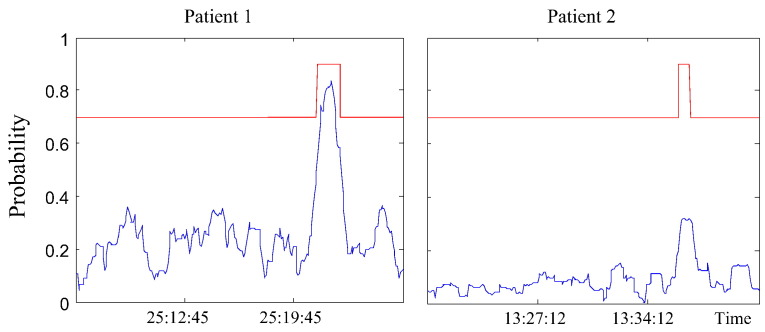
An example of the probabilistic output of the system for two fifteen-minute EEG segments from 2 patients. Clinical annotations are superimposed on top in red.

Fig. 5 shows an example of the probabilistic output for 2 patients for 15-minute EEG segments. In both cases, an increase in the probability was seen for a seizure event. Although, both probabilistic traces show perceivable difference between the probabilistic levels for seizure and non-seizure, it is obvious that the AUC computed by averaging the sensitivity and specificity over a set of common fixed thresholds across the two patients will be lower than the average of the AUCs computed for each patient independently. Hence, if the system output is supposed to be visualized using the probabilistic trace (or the colormap), the correct way to report the performance of such a system is to compute the AUC from each patient and then average.

## 5 Survey results and discussion

To determine the most suitable, convenient and synergetic way to visualize the output of the developed seizure detector in a clinical environment, a survey of the clinical personnel from the NICU of Cork University Maternity Hospital was performed and is reported in this study. Fourteen people participated in the survey. Among them there were 5 neonatologists with experience in interpreting EEG. They directly represent potential end users of the DSS. Their opinions are the most valuable for the scope of the study and are presented here.

**Fig. 6:**
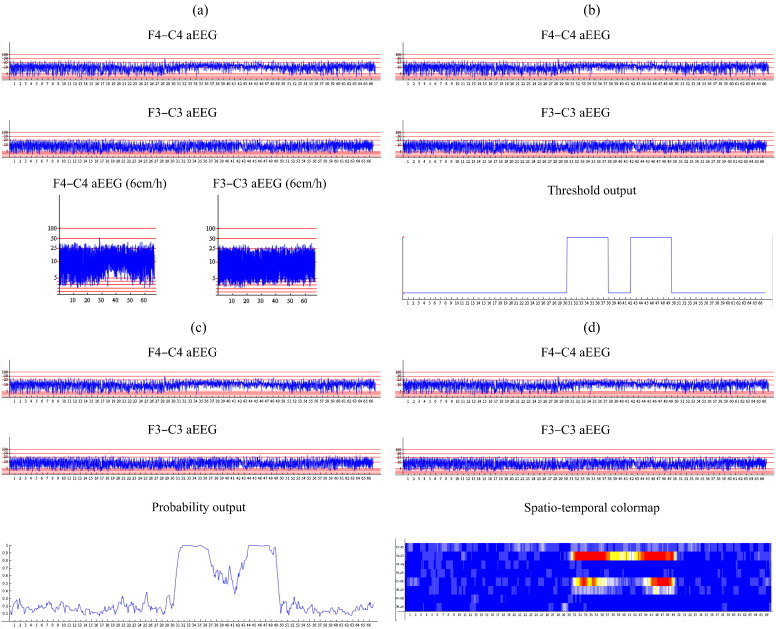
An example of the 4 slides made for each of the 9 test examples, (a) aEEG alone, (b) aEEG + binary, (c) aEEG + probabilistic, and (d) aEEG + spatio-temporal colormap.

The survey was organized as follows. Eleven 1 h 8-channel EEG segments were selected from the database of continuous neonatal EEG [19,21]. For each EEG segment, 4 slides were made as shown in the example in Fig. 6. The first slide is aEEG only which represents current clinical practice. The other slides show a combination of the current clinical practice with the output of the DSS; aEEG + binary, aEEG + probabilistic, and aEEG + spatio-temporal colormap. In total, 44 slides were created. Two examples, one with no seizures and the other with a single clear 5-minute-long seizure in the middle, were used for quick training purposes. This is done in order to explain to the surveyed audience how the output of the classifier typically looks for seizure and background EEG in the form of binary, probabilistic and spatio-temporal colormap output. The remaining 9 examples formed 36 slides which were proposed to the audience. The order of slides was randomised to eliminate possible effects of learning during survey. It was assured that the audience could not change their previous decisions, e.g. when accidentally the more informative system output such as the colormap followed the binary output for the same example. The audience was instructed that some examples may contain no seizures.

For every slide, the audience was asked 3 questions: 1) ‘Is there a seizure in the recording?’ 2) ‘How many seizures are there?’, and 3) ‘Provide time onset and offset of every detected seizure’. These questions target 3 levels of metrics: patient based, event-based and epoch-base as discussed in Section 4. The first question intends to capture whether a baby with at least one seizure has been missed. The second one identifies whether or not all of the seizures were caught. The third question allows for computation of the number of false seizures detected and the temporal accuracy of the detected seizure. At the end of the survey, the audience was asked which visualization technique was found more appropriate, useful, or convenient. In total, the audience was asked 109 questions (36 ∗ 3 + 1).

For EEG audification, a similar survey was performed online where the audified EEG output was accompanied with the corresponding aEEG traces. The audience was given the same 11 EEG segments (2 for training and 9 for testing) which were 6 min of audio per an hour of EEG. All examples of audified neonatal EEG used in the survey can be found online, http://rennes.ucc.ie/~andreyt/visual/. Neither neonatal clinicians nor other surveyed users had any experience in listening to audified EEG (some have ‘listened’ to old paper-based EEG machines). For this reason, the results of audified EEG presented here are obtained from the surveyed audience including non-healthcare professionals.

With respect to the first two questions of the survey, aEEG and the 3 visualization methods each provided 100% accuracy in identification of the non-seizure examples. With regards to the seizure examples, the audience using aEEG alone identified only 67% of recordings with at least 1 seizure. It is slightly higher than the sensitivity reported in the literature. For instance, in [43] using aEEG 57% of the seizure-containing records were detected with no false-positive seizure detections in control records. The performance of aEEG greatly depends on the experience of the user and may vary significantly. Importantly, all 3 visualization methods increased the ability of a clinician to identify whether a given segment contained at least 1 seizure with 80%, 92%, and 88% accuracy, for binary, probabilistic and colormap, respectively.

**Fig. 7:**
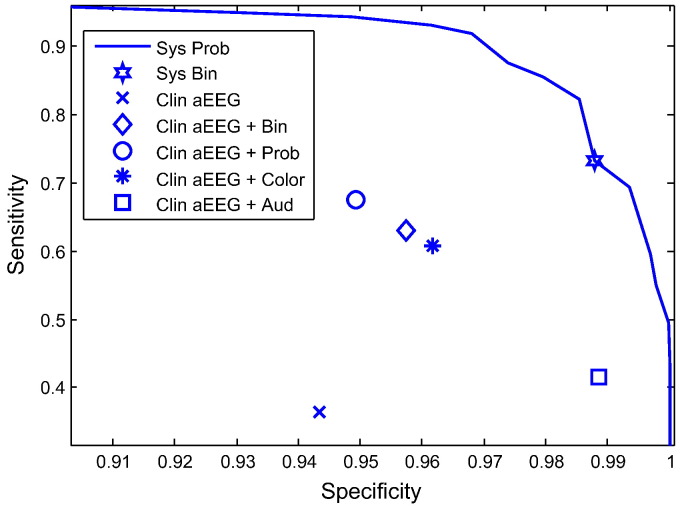
The results of the survey.

The answers to question 3 of the survey are summarised in Fig. 7 for the epoch-based sensitivity and specificity metrics. The ‘Sys Prob’ curve (probabilistic system output) and the ‘Sys Bin’ point (binary system output) indicate the performance of the system itself on the chosen examples. The other points, ‘Clin aEEG’, ‘Clin aEEG + Bin’, ‘Clin aEEG + Prob’, ‘Clin aEEG + Color’, and ‘Clin aEEG + Aud’ indicate the performance achieved by the surveyed audience using aEEG alone, aEEG + binary, aEEG + probabilistic, aEEG + spatio-temporal colormap, and aEEG + audified EEG, respectively.

It can be seen that the performance achieved with aEEG alone conforms to what has been previously reported. Sensitivity of 38% and specificity of 92% using aEEG were reported in [5]. Sensitivity of 12%–38% has been reported in [43]. It can be seen from Fig. 7 that all three visual methods increased the performance of a conventional aEEG diagnosis. The colormap resulted in the highest specificity and the probability method resulted in the highest sensitivity. If the audience had absolute confidence in the system, then of course, all three points would be on the curve. On the contrary, the results of the visualization methods lie between the results of using aEEG alone and the system performance curve. This indicates that clinicians by default trust aEEG and it would take time for them to gain confidence in the algorithm, regardless of which visualization method is eventually selected. Interestingly, the binary output is slightly closer to the colormap than to the probabilistic output.

It can also be observed from Fig. 7 that the results of using audified EEG are separated from all other methods. It has been observed that certain seizure morphologies resulted in a very distinct high pitched sound. This technique provided the second lowest sensitivity which indicates that not all seizures resulted in this sound. However, this clearly audible high pitched phenomenon was seen to be solely specific to seizures as indicated by its specificity which is by far the largest among all the considered methods.

It is worth noting that the location of the points which resulted from the survey should be compared to each other rather than to the system performance curve. The curve of system performance per se depends on the complexity of the 9 chosen examples. In this study, the performance equals to 98% of AUC, which is larger than the AUC of 95–97% previously reported for the same system [20,34]. This left little space for the surveyed audience to improve over the system results.

The results of visualization methods along with the system validation results indicate that the developed neonatal seizure detector with its current level of performance would unambiguously be of benefit to clinicians as a DSS and will increase the neonatal seizure detection rate.

## 6 The interface of the DSS for the clinical trial

**Fig. 8:**
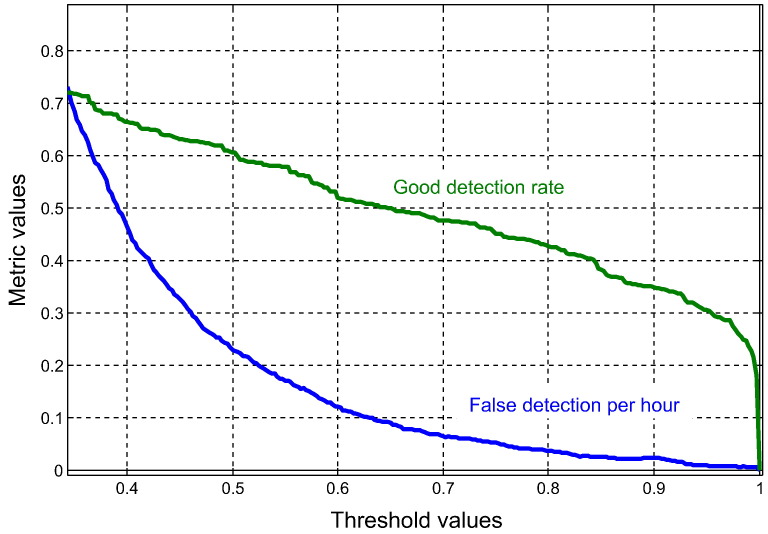
Threshold selection guide.

Answering the very last question of the survey, 3 out of 5 clinicians named the binary system output to be the most convenient. As discussed in Section 3, the binary output needs a threshold to be defined. For this reason, the dependency of the system performance on the threshold selection was investigated. The two metrics considered were the good detection rate which is the percentage of correctly detected seizures and the number of false detections per hour. The former defines the event sensitivity of the algorithm and the latter indicates the cost. Fig. 8 shows how both the good detection rate and the number of false detections per hour decrease by increasing the threshold on seizure probability. It should be noted that Fig. 8 plots the average seizure detection rate and the upper bound of the 95% confidence interval of the number of false detections per hour. As such the performance in Fig. 8 is over-pessimistic, as it displays the regular benefits at the worst-case-scenario cost. It can be seen from Fig. 8 that the system can detect 50% of seizures with a cost bounded by 1 false alarm every 10 h (threshold = 0.65). Alternatively, 30% of seizures can be detected with a cost bounded by 1 false alarm every 100 h (threshold = 0.95) or at a cost of 1 false alarm every 5 h, the system can detect 60% of seizures. From Fig. 8 a threshold of 0.5 was agreed to be fixed throughout the clinical trial.

**Fig. 9:**
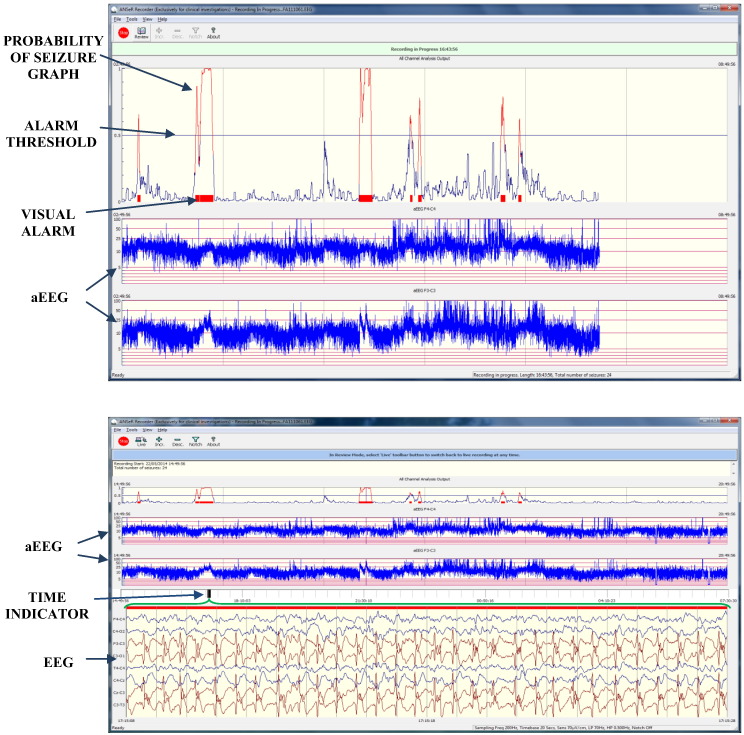
Top: The real-time interface of the decision support system for the clinical trial. Bottom: The review mode.

Fig. 9 (top) shows the interface of the DSS which is currently undergoing pre-market European multi-centre clinical investigation to support its regulatory approval and clinical adoption (the ANSeR study—Algorithm for Neonatal Seizure Recognition http://clinicaltrials.gov/show/NCT02160171). The output of the decision support tool was chosen to be a combination of the binary and probabilistic outputs of the classifier. The upper most trace displays the probabilistic output which is plotted in blue when it is below the threshold and in red when it surpasses the threshold and complies with the artefact detector [33]. Below this, the tool also shows 2 channels of aEEG. The tool allows for clicking on the probabilistic trace or the aEEG trace, in which case the review pane is opened as shown in Fig. 9 (bottom). The time indicator shows the chosen time-point for reviewing. The multi-channel EEG activity that corresponds to that time point as indicated by the green brace is displayed. This interface has been agreed by the participants of the clinical trial which represent 8 maternity hospitals around Europe.

**Fig. 10:**
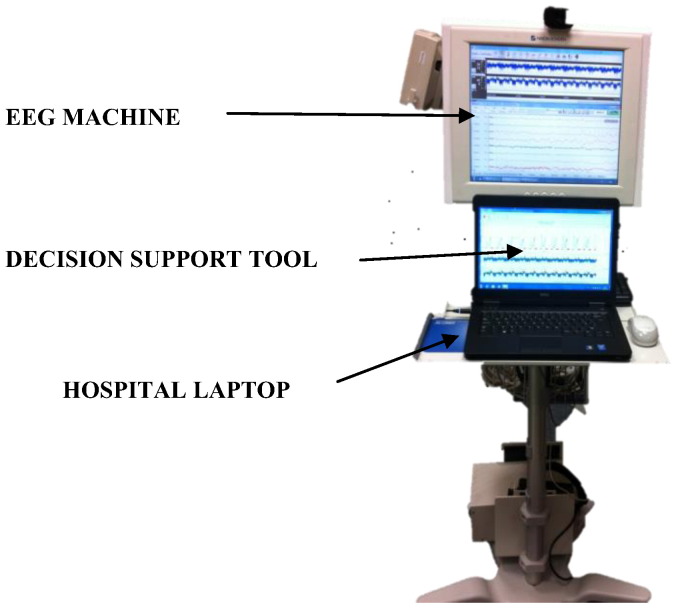
Decision support tool architecture.

Fig. 10 shows the architecture of the DSS in a clinical environment. The software system requires the EEG acquisition system for operation. The DSS is installed on a laptop connected to the EEG acquisition system.

## 7 Relation to the theory of decision support system

A clinical DSS is defined as interactive computer software which is designed to assist healthcare professionals in the decision making process [49,57]. There exist a number of taxonomies that can describe a given DSS [50]. The system presented in this work is an *active intelligent single-user DSS* that is capable of bringing out explicit suggestions by using elements of artificial intelligence.

Although clinical DSS have shown great promise in reducing medical errors and improving patient care they do not always result in improved clinical practice, for reasons that are not always clear. The importance of rigorous and adaptive clinical DSS design to bridge the gap between academic research and clinical practice using technology acceptance models has been discussed in [53]. The concepts of acceptance behaviour formation and actual system use were incorporated into the existing technology acceptance model. The model of unified theory of acceptance and use of technology has also been used in [54]. The strategies to facilitate more widespread technology adoption were identified. The main factors that influence DSS acceptance and use were the DSS usefulness, trust in the knowledge base, presentation of information, ease of use and facilitating conditions (workflow integration). The key experiences from previous efforts to design and implement clinical DSS have also been summarised in [58]. The quality and timeliness of information provided by the developed DSS have been tackled by the rigorous technical validation [19,33] and will be addressed in further studies of the group in the evidence from the clinical trial.

It is true that the pathway to clinical adoption of any DSS is partly hindered by the unnecessary workflow disruptions introduced [57]. The importance of integrating a newly developed DSS into the established clinical workflow has been stressed in many studies [49,51]. Analysis of 70 randomised controlled trials reported in [52] has concluded that an effective clinical DSS must minimise the effort required by clinicians to receive and act on system recommendations. Given that the demand on staff time is high, the DSS must become a fluid and integral part of the workflow. In this work the developed DSS is presented on the laptop along with the current clinical practice monitors. This decision was driven by the regulatory constraints. Ultimately, the DSS system will be incorporated into the EEG software system. The clinician does not have to stop working on the existing information systems in order to access the DSS recommendations. Additionally, the data are fed to the developed software from the same recording device so that the information provided by the current clinical practice and the DSS recommendations are synchronised in time.

The remaining concepts to be addressed are the ease of use and presentation of information of the DSS. The ‘five rights’ of the clinical DSS are known as right information to the right people through the right channel *in the right form* and at the right time [58]. In other words, the key questions are those whose decisions are being supported, what information is presented, when it is presented and how it is presented [57]. The form that the information is presented in may potentially convert an important DSS to an unusable and redundant piece of software. Information visualization aims to achieve several goals such as intuitive data formatting, emphasising subtle aspects of reasoning, or to prevent information overload. The latter, the problem of very dense display of data in the context of intensive cares units while monitoring patients with severe brain injury has been addressed in [55] including temporal data abstraction, principal component analysis, and clustering methods. A number of intelligent information visualization methods have been surveyed in [56]. A categorization scheme was developed based on representation criteria such as 2D vs 3D, static vs dynamic, with the application to time-series analysis. Recent works on the intelligent visualization and interpretation of clinical data have been reviewed in [61]. Information visualization is closely related to the decision making biases. It has been argued that error management theory may explain the evolution of cognitive and behavioural biases in human decision making under uncertainty [59]. Traditional and novel technology-mediated medical decision making approaches have been critically examined in [60]. This study quantitatively and qualitatively addresses the problem of information representation. Information provided by the DSS extends natural human cognitive limitations by using visual and auditory systems. In this manner, the load imposed by information gathering goals can be alleviated to allocate more cognitive resources for discriminating among hypothesis and making complex decisions [60].

## 8 Healthcare benefits

The transformation of neonatal care over the last 20 years has resulted in extremely premature and very ill babies having a better chance of survival than ever before. However it is still difficult to predict which babies will die and which will survive with severe disabilities. The social consequences and lifelong economic costs resulting from neonatal brain injury are extremely high. The challenge for modern medicine is to reduce the disability rate by understanding which factors cause these problems, how to detect and treat them early and how to prevent them. Neonatal seizures are a common emergency in intensive care, occurring in about 1–3 per 1000 babies born at term (they are more common in preterm babies, [45]). To put this in perspective, the number of births in Ireland is approximately 75,000 per year, in the UK, 700,000 and worldwide there are approximately 131 million births each year.

Hypoxic ischaemic encephalopathy as a result of perinatal asphyxia is the commonest cause of seizures in neonates and represents a very significant health-care and financial burden. Globally, this is a much larger problem with 23% of the 4 million neonatal deaths worldwide being due to perinatal asphyxia [46]. The risk of permanent neurological injury causing lifelong disability after HIE complicated by seizures is high, and the costs of care for disabled survivors is usually several million dollars. NICU costs in the UK are estimated at over $2000 per day, with an average duration of admission of 10 days. Estimated costs of disabled children range from $30,000–$120,000 yearly for moderately and severely disabled children respectively. The disability experienced by survivors includes cerebral palsy, epilepsy and learning difficulties [47]. The quality of life of the child with profound neurological handicap is very poor. The amount of care which disabled children require has implications for parents, siblings and the health service. Improvement of neurodevelopmental outcome could have a dramatic impact on these children and their families.

On the other hand, over-treatment of babies with antiepileptic drugs carries the risk of using neurotoxic medications, prolonging intensive care (with associated costs and parental separation) and increases the risk of complications. The current clinical standard of care is to treat babies based solely on clinical diagnosis of seizures (physical manifestations). EEG studies carried out by ourselves and others have shown this to be inaccurate and unreliable and to lack any evidence base [48]. Increasingly, clinicians are using cot-side aEEG to guide their therapy but surveys show that interpretation skills are limited, and this method does not reliably detect all seizures [3]. Clinicians caring for babies affected by seizures are poorly supported by specialist neurophysiology, which is a scarce resource, and would embrace and welcome an intelligent cot-side decision support tool. A robust, reliable, automated seizure detection system which is easy to use and interpret would be widely welcomed. Such a system would ensure prompt recognition of ‘true’ seizures and facilitate individual tailoring of antiepileptic drug treatment, avoiding prolonged multi-drug regimens. This should improve neurodevelopmental outcome and reduce intensive care days. In addition, babies with jittery movement patterns which are not epileptic would quickly be recognised as ‘non-seizure’ and would avoid invasive investigations, separation from their parents, and unnecessary intensive care admissions involving treatment with potentially toxic drugs.

## 9 Future work

The developed neonatal seizure detection algorithm will be the first to be tested in a randomised clinical trial. There are a number of trial outcomes that will have to be further analysed and will form part of our future work.

Previous studies in the area have discussed a number of different metrics which are summarised in Section 4. These metrics range from purely engineering, signal processing and machine learning perspectives to more clinical viewpoints. However, the evaluation setup and metrics have implicitly considered an ‘offline’ scenario. In contrast, the ‘online’ scenario, that is running a tool not retrospectively but in a real clinical setting, may have different milestones. To date there has been little work done on connecting the reported metrics to real-life effects. The improvement of the neurodevelopmental outcome of the babies is a final target which has a number of constituents; for instance, the number of antiepileptic drugs given correctly or in vain, time points of these drugs relative to the onset of seizures, etc. These metrics will be back traced to the original mathematical formulations of the decision support system and may result in a number of important changes. For instance, it may be beneficial to have a higher confidence when detecting the very first seizure or detection of longer seizures may be prioritised. These open questions will have to be answered.

The level of agreement between the annotations (inter-observer agreement) and the neonatal seizure detection algorithm will be also assessed using a variety of measures. It will allow for comparison of the level of accuracy of the algorithm with that of human expert error.

## 10 Conclusions

Three different visualization methods to convey information from the developed neonatal seizure detection system have been presented, discussed and contrasted. Their relation to the metric computation methods has been established. The algorithm-driven audification of neonatal EEG has also been explored as an alternative to visual aids. A survey of the targeted end users was made in order to determine the level of optimality of each of the proposed methods. It has been shown that all methods have the potential to improve the performance of neonatal seizure detection in a clinical environment over the conventional aEEG approach. Without any dedicated training of clinical personnel, the binary visualization form was preferable. The survey results have assisted in the definition of the decision support tool interface. The decision support tool with the chosen visualization interface is currently undergoing pre-market European multi-centre clinical investigation to support its regulatory approval and clinical adoption.

## References

[1] Rennie J., Boylan G. (2007). Treatment of neonatal seizures. Archives of Disease in Childhood.

[2] Murray D., Boylan G., Ali I., Ryan C., Murphy B., Connoly S. (2008). Defining the gap between electrographic seizure burden, clinical expression and staff recognition of neonatal seizures. Archives of Disease in Childhood.

[3] Boylan G., Burgoyne L., Moore C., O'Flaherty B., Rennie J. (2010). An international survey of EEG use in the neonatal intensive care unit. Acta Paediatrica.

[4] Toet M., Lemmers P. (2009). Brain monitoring in neonates. Early Human Development.

[5] Rennie J., Chorley G., Boylan G., Pressler R., Nguyen Y., Hooper R. (2004). Non-expert use of the cerebral function monitor for neonatal seizure detection. Archives of Disease in Childhood — Fetal and Neonatal Edition.

[6] Celka P., Colditz P. (2002). A computer-aided detection of EEG seizures in infants, a singular-spectrum approach and performance comparison. IEEE Transactions on Biomedical Engineering.

[7] Liu A., Hahn J., Heldt G., Coen R. (1999). Detection of neonatal seizures through computerized EEG analysis. Electroencephalography and Clinical Neurophysiology.

[8] Gotman J., Flanagan D., Zhang J., Rosenblatt B. (1997). Automatic seizure detection in the newborn: methods and initial evaluation. Electroencephalography and Clinical Neurophysiology.

[9] Roessgen M., Zoubir A., Boashash B. (1998). Seizure detection of newborn EEG using a model-based approach. IEEE Transactions on Biomedical Engineering.

[10] Deburchgraeve W., Cherian P., de Vos M., Swarte R., Blok J., Visser G., Govaert P., Van Huffel S. (2008). Automated neonatal seizure detection mimicking a human observer reading EEG. Clinical Neurophysiology.

[11] Aarabi A., Grebe R., Wallois F. (2007). A multistage knowledge-based system for EEG seizure detection in newborn infants. Clinical Neurophysiology.

[12] Cherian P., Deburchgraeve W., Swarte R., De Vos M., Govaert P., Van Huffel S., Visser G. (2011). Validation of a new automated neonatal seizure detection system: a clinician's perspective. Clinical Neurophysiology.

[13] Mitra J., Glover J., Ktonas P., Kumar A., Mukherjee A., Karayiannis N., Frost J., Hrachovy R., Mizrahi E. (2009). A multistage system for the automated detection of epileptic seizures in neonatal electroencephalography. Journal of Clinical Neurophysiology.

[14] Navakatikyan M., Colditz P., Burke C., Inderd T., Richmond J., Williams C. (2006). Seizure detection algorithm for neonates based on wave-sequence analysis. Clinical Neurophysiology.

[15] Smit L., Vermeulen R., Fetter W., Strijers R., Stam C. (2004). Neonatal seizure monitoring using non-linear EEG analysis. Neuropediatrics.

[16] Stevenson N., O'Toole J., Rankine L., Boylan G., Boashash B. (2012). A nonparametric feature for neonatal EEG seizure detection based on a representation of pseudo-periodicity. Medical Engineering and Physics.

[17] Faul S., Boylan G., Connolly S., Marnane W., Lightbody G. (2005). An evaluation of automated neonatal seizure detection methods. Clinical Neurophysiology.

[18] Mitchell M. (1997). Machine Learning.

[19] Temko A., Thomas E., Marnane W., Lightbody G., Boylan G. (2011). EEG-based neonatal seizure detection with support vector machines. Clinical Neurophysiology.

[20] Temko A., Thomas E., Marnane W., Lightbody G., Boylan G. (2011). Performance assessment for EEG-based neonatal seizure detectors. Clinical Neurophysiology.

[21] Temko A., Stevenson N., Marnane W., Boylan G., Lightbody G. (2012). Inclusion of temporal priors for automated neonatal EEG classification. Journal of Neural Engineering.

[22] S. Faul, A. Temko, W. Marnane, G. Lightbody, and G. Boylan, A Method for the Real-time Identification of Seizures in an Electroencephalogram (EEG) Signal, patent ID: WO/2010/115939, 2010.

[23] Wagholikar K., Sundararajan V., Deshpande A. (2012). Modeling paradigms for medical diagnostic decision support: a survey and future directions. Journal of Medical Systems.

[24] Copetti A., Leite J.C.B., Loques O., Neves M. (2013). A decision-making mechanism for context inference in pervasive healthcare environments. Decision Support Systems.

[25] Naderpour M., Lu J., Zhang G. (2014). An intelligent situation awareness support system for safety-critical environments. Decision Support Systems.

[26] Kitayama M., Otsubo H., Parvez S., Lodha A., Ying E., Parvez B., Ishii R., Mizuno-Matsumoto Y., Zoroofi R., Snead O. (2003). Wavelet analysis for neonatal electroencephalographic seizures. Pediatric Neurology.

[27] De Weerd A., Despland P., Plouin P. (1999). Neonatal EEG. The International Federation of Clinical Neurophysiology. Electroencephalography and Clinical Neurophysiology. Supplement.

[28] Platt J. (1999). Probabilistic outputs for SVM and comparison to regularized likelihood methods. Advances in Large Margin Classifiers.

[29] Temko A., Nadeu C., Marnane W., Boylan G., Lightbody G. (2011). EEG signal description with spectral-envelope-based speech recognition features for detection of neonatal seizures. IEEE Transactions on Information Technology in Biomedicine.

[30] Thomas E., Temko A., Lightbody G., Marnane W., Boylan G. (2010). Gaussian mixture models for classification of neonatal seizures using EEG. Physiological Measurement.

[31] Thomas E., Temko A., Marnane W., Boylan G., Lightbody G. (2013). Discriminative and generative classification techniques applied to automated neonatal seizure detection. IEEE Journal of Biomedical and Health Informatics.

[32] Temko A., Lightbody G., Thomas E., Boylan G., Marnane W. (2012). Instantaneous measure of EEG channel importance for improved patient-adaptive neonatal seizure detection. IEEE Transactions on Biomedical Engineering.

[33] Temko A., Boylan G., Marnane W., Lightbody G. (2013). Robust neonatal EEG seizure detection through adaptive background modelling. International Journal of Neural Systems.

[34] Low E., Stevenson N., Temko A., Lightbody G., Marnane W., Livingstone V., Mathieson S., Ryan C., Rennie J., Boylan G. (2011). Clinical validation of a neonatal seizure detection algorithm. Pediatric Research.

[35] El-Dib M., Chang T., Tsuchida T., Clancy R. (2009). Amplitude-integrated electroencephalography in neonates. Pediatric Neurology.

[36] McNeil B., Keller E., Adelstein S. (1975). Primer on certain elements of medical decision making. New England Journal of Medicine.

[37] Baier G., Hermann T., Stephani U. (2007). Event-based sonification of EEG rhythms in real time. Clinical Neurophysiology.

[38] Khamis H., Mohamed A., Simpson S., McEwan A. (2012). Detection of temporal lobe seizures and identification of lateralisation from audified EEG. Clinical Neurophysiology.

[39] Ellis D. A Phase Vocoder in Matlab, Lab for Recognition and Organization of Speech and Audio. http://www.ee.columbia.edu/~dpwe/resources/matlab/pvoc/.

[40] Portnoff M. (1976). Implementation of the digital phase vocoder using the Fast Fourier Transform. IEEE Transactions on Acoustics, Speech, and Signal Processing.

[41] Logesparan L., Casson A., Rodriguez-Villegas E. (2011). Performance metrics for characterization of a seizure detection algorithm for offline and online use. 5th International Workshop on Seizure Prediction, Dresden, Germany.

[42] Freeman J. (2007). The use of amplitude-integrated electroencephalography: beware of its unintended consequences. Pediatrics.

[43] Shellhaas R., Soaita A., Clancy R. (2007). Sensitivity of amplitude-integrated electroencephalography for neonatal seizure detection. Pediatrics.

[44] Vanhatalo S. (2011). Development of neonatal seizure detectors: an elusive target and stretching measuring tapes. Clinical Neurophysiology.

[45] Glass H., Pham T., Danielsen B., Towner D., Glidden D., Wu Y. (2009). Antenatal and intrapartum risk factors for seizures in term newborns: a population-based study. Journal of Pediatrics.

[46] Lawn J., Cousens S., Zupan J. (2005). Neonatal survival 1–4 million neonatal deaths: When? Where? Why?. Lancet.

[47] Mwaniki M., Atieno M., Lawn J., Newton C. (2012). Long-term neurodevelopmental outcomes after intrauterine and neonatal insults: a systematic review. Lancet.

[48] Rennie J., Chorley G., Boylan G., Pressler R., Nguyen Y., Hooper R. (2004). Non-expert use of the cerebral function monitor for neonatal seizure detection. Archives of Disease in Childhood — Fetal and Neonatal Edition.

[49] Yao W., Kumar A. (2014). CONFlexFlow: integrating flexible clinical pathways into clinical decision support systems using context and rules. Decision Support Systems.

[50] Power D. (2002). Decision Support Systems: Concepts and Resources for Managers.

[51] Karsh B. (2009). Clinical practice improvement and redesign: how change in workflow can be supported by clinical decision support. AHRQ Publication No. 09-0054-EF.

[52] Kawamoto K., Houlihan C., Balas E., Lobach D. (2005). Improving clinical practice using clinical decision support systems: a systematic review of trials to identify features critical to success. BMJ.

[53] Johnson M., Zheng K., Padman R. (2014). Modeling the longitudinality of user acceptance of technology with an evidence-adaptive clinical decision support system. Decision Support Systems.

[54] Shibl R., Lawley M., Debuse J. (2013). Factors influencing decision support system acceptance. Decision Support Systems.

[55] Kamsu-Foguem B., Tchuenté-Foguem G., Allart L., Zennir Y., Vilhelm C., Mehdaoui H., Zitouni D., Hubert H., Lemdani M., Ravaux P. (2012). User-centered visual analysis using a hybrid reasoning architecture for intensive care units. Decision Support Systems.

[56] Aigner W., Miksch S., Müller W., Schumann H., Tominski C. (2007). Visualizing time-oriented data — a systematic view. Computers and Graphics.

[57] Berner E. (2009). Clinical decision support systems: State of the art, Agency for Healthcare Research and Quality; Rockville. AHRQ, Publication No. 09–0069-EF.

[58] Handler S., Sharkey S., Hudak S., Ouslander J. (2011). Incorporating INTERACT II clinical decision support tools into nursing home health information technology. Annals of Longterm Care.

[59] Johnson D., Blumstein D., Fowler J., Haselton M. (2013). The evolution of error: error management, cognitive constraints, and adaptive decision-making biases. Trends in Ecology & Evolution.

[60] Patel V., Kaufman D., Arocha J. (2002). Emerging paradigms of cognition in medical decision-making. Journal of Biomedical Informatics.

[61] Klimov D., Shahar Y., Taieb-Maimon M. (2010). Intelligent querying, visualization, and exploration of the time-oriented data of multiple patients. Artificial Intelligence in Medicine.

